# (*Z*)-4-[3-(2,5-Dioxoimidazolidin-4-ylidenemeth­yl)-1*H*-indol-1-ylmeth­yl]benzonitrile

**DOI:** 10.1107/S1600536808032601

**Published:** 2008-10-18

**Authors:** Narsimha Reddy Penthala, Thirupathi Reddy Yerram Reddy, Sean Parkin, Peter A. Crooks

**Affiliations:** a Department of Pharmaceutical Sciences, College of Pharmacy, University of Kentucky, Lexington, KY 40536, USA; b Department of Chemistry, University of Kentucky, Lexington, KY 40506, USA

## Abstract

In the title compound, C_20_H_14_N_4_O_2_, mol­ecules are linked into chains by N—H⋯O hydrogen bonds, but the cyano group does not participate in the supra­molecular aggregation. The crystal structure of the compound indicates the presence of a double bond with *Z* geometry, connecting the imidazolidine and indole units. The dihedral angle between the imidazole and benzene ring planes is 62.45 (4)°.

## Related literature

For 2-indol-3-yl-methyl­enequinuclidin-3-ols NADPH oxidase activity, see: Sekhar *et al.* (2003 ▶). For novel substituted (*Z*)-2-(*N*-benzyl­indol-3-ylmethyl­ene)quinuclidin-3-one and (*Z*)-(±)-2-(*N*-benzyl­indol-3-ylmethyl­ene)quinuclidin-3-ol derivatives as potent thermal sensitizing agents, see: Sonar *et al.* (2007 ▶). For the mol­ecular structures of di- and triindolyl­methanes, see: Mason *et al.* (2003 ▶). For the structures of 1*H*-indole-3-ethyl­ene-3′-methoxy­salicylaldimine and 3-[3′-aza­pentyl-3′-en-4′-(2′′-hydroxy­phen­yl)]indole, see: Zarza *et al.* (1988 ▶).
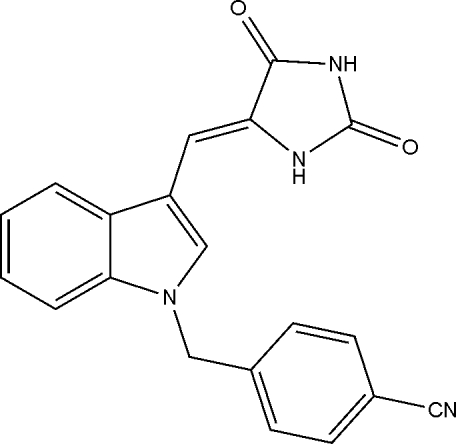


## Experimental

### 

#### Crystal data

C_20_H_14_N_4_O_2_
*M* *_r_* = 342.35
Monoclinic, 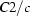

*a* = 18.8495 (16) Å
*b* = 7.6812 (7) Å
*c* = 24.322 (2) Å
β = 110.939 (3)°
*V* = 3289.0 (5) Å^3^
*Z* = 8
Cu *K*α radiation
μ = 0.76 mm^−1^
*T* = 90.0 (2) K
0.15 × 0.08 × 0.06 mm

#### Data collection

Bruker X8 Proteum diffractometer
Absorption correction: multi-scan (*SADABS*; Bruker, 2006 ▶) *T*_min_ = 0.806, *T*_max_ = 0.957
23493 measured reflections
3025 independent reflections
2849 reflections with *I* > 2σ(*I*)
*R*_int_ = 0.039

#### Refinement

*R*[*F* ^2^ > 2σ(*F* ^2^)] = 0.034
*wR*(*F* ^2^) = 0.088
*S* = 1.04
3025 reflections
236 parameters
H-atom parameters constrained
Δρ_max_ = 0.24 e Å^−3^
Δρ_min_ = −0.30 e Å^−3^

### 

Data collection: *APEX2* (Bruker, 2006 ▶); cell refinement: *SAINT* (Bruker, 2006 ▶); data reduction: *SAINT*; program(s) used to solve structure: *SHELXS97* (Sheldrick, 2008 ▶); program(s) used to refine structure: *SHELXL97* (Sheldrick, 2008 ▶); molecular graphics: *XP* in *SHELXTL* (Sheldrick, 2008 ▶); software used to prepare material for publication: *SHELXL97* and local procedures.

## Supplementary Material

Crystal structure: contains datablocks global, I. DOI: 10.1107/S1600536808032601/fj2156sup1.cif

Structure factors: contains datablocks I. DOI: 10.1107/S1600536808032601/fj2156Isup2.hkl

Additional supplementary materials:  crystallographic information; 3D view; checkCIF report

## Figures and Tables

**Table 1:** Hydrogen-bond geometry (Å, °)

*D*—H⋯*A*	*D*—H	H⋯*A*	*D*⋯*A*	*D*—H⋯*A*
N3—H3⋯O1^i^	0.88	1.95	2.8237 (12)	173
N4—H4⋯O2^ii^	0.88	2.07	2.8740 (13)	151

Symmetry codes: (i) 
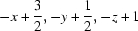
; (ii) 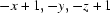.

**Fig. 1.**
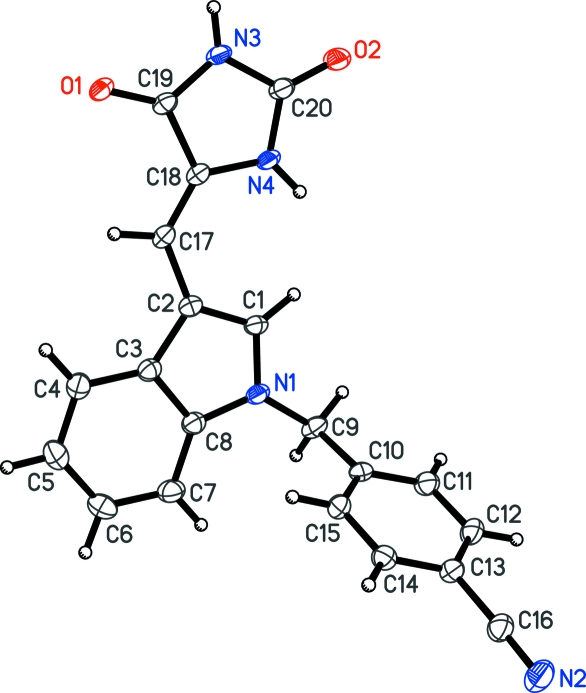
A view of the molecule with the atom numbering scheme. Displacement ellipsoids are drawn at the 50% probability level.

## supplementary crystallographic information

### Comment

As part of a continuing search for biologically active molecules containing indole ring systems (Sonar *et al.*, 2007), we have now prepared the title compound by the reaction of 4-(3-formyl-1*H*-indol-1-ylmethyl)benzonitrile with imidazolidine-2,4-dione in the presence of ammonium acetate in acetic acid at 393 K. The compound was crystallized from a mixture of methanol and ethylacetate. The molecular structure and the atom-numbering scheme are shown in Fig.1. The indole ring is planar with bond distances and angles comparable with those previously reported for other indole derivatives (Mason *et al.*, 2003; Zarza, *et al.*, 1988). The X-ray studies revealed that the obtained compound is the *Z* isomer. The C18—C19 bond is in a *trans* position with respect to the C2—C17 bond. The olefinic bond (C17=C18) has a planar atomic arrangement, since the r.m.s. deviation from the mean plane passing through atoms C2, C17, C18, N4 is 0.0009 (5) Å. The maximum deviation from plane for imidazoline ring is 0.0087 Å. Deviations from ideal geometry are observed in the bond angles around atoms C2, C17 and C18. The C17=C18—C19 bond angle is close to the standard planar triangular value of 120°, whereas the C1=C2—C17, C18—C17=C2 and C17=C18—N4 bond angles are more distorted due to the strain induced by the C17=C18—C1=O1 conjugated double bond linkage. These bond angle deformations, which require little energy, are needed to release the intramolecular interactions between non-bonded atoms. The imidazolidine ring, which makes a dihedral angle of 2.48 (5)° with the adjacent aromatic ring presents very small distortions around atoms N4, C20, N3 and C19.

Significant intermolecular hydrogen-bonding interactions are found between N(3)—H(3)···O(1) and N(4)—H(4)···O(2). Molecules are linked into chains by a series of N—H···O hydrogen bonds.

### Experimental

A mixture of 4-(3-formyl-indol-1-ylmethyl)benzonitrile (0.5 g, 1.92 mmol), imidazolidine-2,4-dione (0.23 g, 2.30 mmol) and ammonium acetate (0.15 g, 1.94 mmol) was stirred in acetic acid (5 ml) at 393 K for 10 hrs. The reaction mixture was cooled to room temperature and the yellow solid that separated was collected by filtration, washed with cold water and dried to afford the the crude product. Crystallization from methanol and ethyl acetate (1:1) gave a yellow crystalline product of (*Z*)-4-((3-((2,5- dioxoimidazolidin-4-ylidene)methyl)-1*H*-indol-1-yl)methyl) benzonitrile that was suitable for X-ray analysis. ^1^H NMR (DMSO d_6_): δ 5.56 (*s*, 2H), 6.7 (*s*, 1H), 7.13–7.23 (*m*, 2H), 7.44–7.53 (*m*, 3H), 7.79–7.83 (*m*, 3H), 8.30 (*s*, 1H), 10.15 (*bs*, 1H), 11.07 (*bs*, 1H); ^13^C NMR (DMSO d_6_): δ 101.46, 109.25, 111.06, 111.22, 119.21, 119.29, 121.34, 123.42, 124.95, 128.10, 128.70, 130.63, 133.26, 136.25, 143.71, 155.94, 165.87.

### Refinement

H atoms were found in difference Fourier maps and subsequently placed in idealized positions with constrained distances of 0.99 Å (*R*_2_CH_2_), 0.95 Å (*R*_2_CH) and 0.88 Å (NH) with *U*_iso_(H) values set to 1.2*U*_eq_ of the attached atom.

### Crystal data

**Table:**

C_20_H_14_N_4_O_2_	*F*(000) = 1424
*M**_r_* = 342.35	*D*_x_ = 1.383 Mg m^−^^3^
Monoclinic, *C*2/*c*	Cu *K*α radiation, λ = 1.54178 Å
Hall symbol: -C 2yc	Cell parameters from 9981 reflections
*a* = 18.8495 (16) Å	θ = 3.9–68.8°
*b* = 7.6812 (7) Å	µ = 0.76 mm^−^^1^
*c* = 24.322 (2) Å	*T* = 90 K
β = 110.939 (3)°	Block, yellow
*V* = 3289.0 (5) Å^3^	0.15 × 0.08 × 0.06 mm
*Z* = 8	

### Data collection

**Table:**

Bruker X8 Proteum diffractometer	3025 independent reflections
Radiation source: fine-focus rotating anode	2849 reflections with *I* > 2σ(*I*)
graded multilayer optics	*R*_int_ = 0.039
Detector resolution: 18 pixels mm^-1^	θ_max_ = 68.8°, θ_min_ = 3.9°
φ and ω scans	*h* = −22→22
Absorption correction: multi-scan (SADABS in APEX2; Bruker, 2006)	*k* = −9→9
*T*_min_ = 0.806, *T*_max_ = 0.957	*l* = −28→29
23493 measured reflections	

### Refinement

**Table:**

Refinement on *F*^2^	Secondary atom site location: difference Fourier map
Least-squares matrix: full	Hydrogen site location: inferred from neighbouring sites
*R*[*F*^2^ > 2σ(*F*^2^)] = 0.034	H-atom parameters constrained
*wR*(*F*^2^) = 0.088	*w* = 1/[σ^2^(*F*_o_^2^) + (0.043*P*)^2^ + 2.7005*P*] where *P* = (*F*_o_^2^ + 2*F*_c_^2^)/3
*S* = 1.04	(Δ/σ)_max_ = 0.001
3025 reflections	Δρ_max_ = 0.24 e Å^−^^3^
236 parameters	Δρ_min_ = −0.30 e Å^−^^3^
0 restraints	Extinction correction: SHELXL97 (Sheldrick, 2008), Fc^*^=kFc[1+0.001xFc^2^λ^3^/sin(2θ)]^-1/4^
Primary atom site location: structure-invariant direct methods	Extinction coefficient: 0.00030 (6)

### Special details

**Table:**

Geometry. All e.s.d.'s (except the e.s.d. in the dihedral angle between two l.s. planes) are estimated using the full covariance matrix. The cell e.s.d.'s are taken into account individually in the estimation of e.s.d.'s in distances, angles and torsion angles; correlations between e.s.d.'s in cell parameters are only used when they are defined by crystal symmetry. An approximate (isotropic) treatment of cell e.s.d.'s is used for estimating e.s.d.'s involving l.s. planes.
Refinement. Refinement of *F*^2^ against ALL reflections. The weighted *R*-factor *wR* and goodness of fit *S* are based on *F*^2^, conventional *R*-factors *R* are based on *F*, with *F* set to zero for negative *F*^2^. The threshold expression of *F*^2^ > 2σ(*F*^2^) is used only for calculating *R*-factors(gt) *etc*. and is not relevant to the choice of reflections for refinement. *R*-factors based on *F*^2^ are statistically about twice as large as those based on *F*, and *R*- factors based on ALL data will be even larger.

### Fractional atomic coordinates and isotropic or equivalent isotropic displacement parameters (Å^2^)

**Table:**

	*x*	*y*	*z*	*U*_iso_*/*U*_eq_	
N1	0.41106 (5)	0.49108 (13)	0.60476 (4)	0.0204 (2)	
N2	0.28685 (7)	0.12998 (16)	0.84996 (5)	0.0344 (3)	
N3	0.66684 (5)	0.16845 (13)	0.50768 (4)	0.0204 (2)	
H3	0.7010	0.1248	0.4946	0.024*	
N4	0.56545 (5)	0.18829 (13)	0.53212 (4)	0.0213 (2)	
H4	0.5232	0.1612	0.5377	0.026*	
O1	0.71827 (4)	0.44259 (11)	0.53352 (4)	0.0233 (2)	
O2	0.58759 (5)	−0.07063 (12)	0.49259 (4)	0.0300 (2)	
C1	0.46081 (6)	0.41065 (15)	0.58406 (5)	0.0196 (2)	
H1	0.4549	0.2960	0.5684	0.024*	
C2	0.52091 (6)	0.51930 (15)	0.58903 (5)	0.0185 (2)	
C3	0.50688 (6)	0.67773 (15)	0.61526 (5)	0.0201 (2)	
C4	0.54595 (7)	0.83512 (16)	0.63133 (6)	0.0269 (3)	
H4A	0.5928	0.8535	0.6257	0.032*	
C5	0.51500 (8)	0.96293 (17)	0.65546 (7)	0.0343 (3)	
H5	0.5412	1.0703	0.6668	0.041*	
C6	0.44596 (8)	0.93872 (17)	0.66367 (6)	0.0337 (3)	
H6	0.4263	1.0298	0.6805	0.040*	
C7	0.40588 (7)	0.78571 (17)	0.64794 (6)	0.0271 (3)	
H7	0.3587	0.7693	0.6531	0.033*	
C8	0.43762 (6)	0.65602 (15)	0.62406 (5)	0.0210 (3)	
C9	0.33815 (6)	0.41858 (17)	0.60065 (5)	0.0235 (3)	
H9A	0.3245	0.3259	0.5704	0.028*	
H9B	0.2993	0.5112	0.5869	0.028*	
C10	0.33468 (6)	0.34369 (15)	0.65670 (5)	0.0199 (3)	
C11	0.27885 (7)	0.22088 (16)	0.65210 (5)	0.0245 (3)	
H11	0.2487	0.1771	0.6145	0.029*	
C12	0.26669 (7)	0.16176 (16)	0.70142 (6)	0.0264 (3)	
H12	0.2280	0.0786	0.6979	0.032*	
C13	0.31168 (7)	0.22512 (15)	0.75657 (5)	0.0225 (3)	
C14	0.36964 (7)	0.34280 (15)	0.76188 (5)	0.0222 (3)	
H14	0.4013	0.3830	0.7996	0.027*	
C15	0.38107 (6)	0.40105 (15)	0.71200 (5)	0.0216 (3)	
H15	0.4209	0.4809	0.7155	0.026*	
C16	0.29783 (7)	0.17006 (16)	0.80831 (5)	0.0266 (3)	
C17	0.58384 (6)	0.49104 (15)	0.57005 (5)	0.0194 (2)	
H17	0.6177	0.5866	0.5755	0.023*	
C18	0.60159 (6)	0.34914 (15)	0.54582 (5)	0.0186 (2)	
C19	0.66927 (6)	0.33333 (15)	0.52922 (5)	0.0190 (2)	
C20	0.60397 (6)	0.07873 (16)	0.50901 (5)	0.0220 (3)	

### Atomic displacement parameters (Å^2^)

**Table:**

	*U*^11^	*U*^22^	*U*^33^	*U*^12^	*U*^13^	*U*^23^
N1	0.0187 (5)	0.0246 (5)	0.0217 (5)	−0.0014 (4)	0.0117 (4)	−0.0015 (4)
N2	0.0372 (6)	0.0407 (7)	0.0285 (6)	−0.0136 (5)	0.0156 (5)	−0.0019 (5)
N3	0.0167 (5)	0.0266 (5)	0.0216 (5)	−0.0027 (4)	0.0116 (4)	−0.0056 (4)
N4	0.0171 (5)	0.0257 (5)	0.0260 (5)	−0.0045 (4)	0.0137 (4)	−0.0056 (4)
O1	0.0205 (4)	0.0268 (4)	0.0277 (4)	−0.0048 (3)	0.0148 (3)	−0.0036 (3)
O2	0.0242 (4)	0.0301 (5)	0.0419 (5)	−0.0081 (4)	0.0195 (4)	−0.0151 (4)
C1	0.0207 (5)	0.0222 (6)	0.0190 (5)	0.0000 (4)	0.0109 (4)	−0.0021 (4)
C2	0.0182 (5)	0.0215 (6)	0.0168 (5)	0.0010 (4)	0.0076 (4)	0.0007 (4)
C3	0.0206 (6)	0.0215 (6)	0.0198 (5)	0.0015 (4)	0.0091 (4)	0.0025 (4)
C4	0.0268 (6)	0.0223 (6)	0.0354 (7)	−0.0011 (5)	0.0156 (5)	0.0008 (5)
C5	0.0384 (7)	0.0207 (6)	0.0477 (8)	−0.0023 (5)	0.0201 (6)	−0.0046 (6)
C6	0.0387 (8)	0.0240 (7)	0.0440 (8)	0.0065 (5)	0.0215 (6)	−0.0037 (6)
C7	0.0270 (6)	0.0275 (6)	0.0318 (7)	0.0052 (5)	0.0166 (5)	0.0017 (5)
C8	0.0217 (6)	0.0218 (6)	0.0213 (6)	0.0019 (4)	0.0100 (5)	0.0022 (4)
C9	0.0181 (5)	0.0327 (7)	0.0223 (6)	−0.0031 (5)	0.0104 (5)	−0.0015 (5)
C10	0.0180 (5)	0.0214 (6)	0.0237 (6)	0.0021 (4)	0.0115 (5)	−0.0017 (4)
C11	0.0230 (6)	0.0280 (6)	0.0239 (6)	−0.0048 (5)	0.0102 (5)	−0.0050 (5)
C12	0.0255 (6)	0.0272 (6)	0.0296 (6)	−0.0080 (5)	0.0137 (5)	−0.0026 (5)
C13	0.0242 (6)	0.0225 (6)	0.0242 (6)	0.0004 (5)	0.0128 (5)	0.0012 (5)
C14	0.0228 (6)	0.0219 (6)	0.0222 (6)	−0.0005 (4)	0.0083 (5)	−0.0016 (4)
C15	0.0192 (5)	0.0216 (6)	0.0255 (6)	−0.0021 (4)	0.0100 (5)	−0.0005 (5)
C16	0.0270 (6)	0.0277 (6)	0.0267 (6)	−0.0060 (5)	0.0118 (5)	−0.0018 (5)
C17	0.0174 (5)	0.0234 (6)	0.0190 (5)	−0.0025 (4)	0.0083 (4)	0.0006 (4)
C18	0.0163 (5)	0.0238 (6)	0.0171 (5)	−0.0012 (4)	0.0078 (4)	0.0002 (4)
C19	0.0180 (5)	0.0252 (6)	0.0156 (5)	−0.0013 (4)	0.0081 (4)	−0.0006 (4)
C20	0.0186 (5)	0.0275 (6)	0.0225 (6)	−0.0038 (5)	0.0105 (4)	−0.0061 (5)

### Geometric parameters (Å, °)

**Table:**

N1—C1	1.3608 (15)	C6—C7	1.3754 (19)
N1—C8	1.3815 (15)	C6—H6	0.9500
N1—C9	1.4527 (14)	C7—C8	1.3913 (17)
N2—C16	1.1459 (17)	C7—H7	0.9500
N3—C19	1.3650 (15)	C9—C10	1.5025 (16)
N3—C20	1.3812 (15)	C9—H9A	0.9900
N3—H3	0.8800	C9—H9B	0.9900
N4—C20	1.3576 (15)	C10—C11	1.3877 (17)
N4—C18	1.3926 (15)	C10—C15	1.3883 (16)
N4—H4	0.8800	C11—C12	1.3761 (17)
O1—C19	1.2246 (14)	C11—H11	0.9500
O2—C20	1.2178 (15)	C12—C13	1.3938 (17)
C1—C2	1.3772 (16)	C12—H12	0.9500
C1—H1	0.9500	C13—C14	1.3879 (17)
C2—C17	1.4348 (16)	C13—C16	1.4372 (17)
C2—C3	1.4416 (16)	C14—C15	1.3805 (17)
C3—C4	1.3967 (17)	C14—H14	0.9500
C3—C8	1.4064 (16)	C15—H15	0.9500
C4—C5	1.3759 (19)	C17—C18	1.3372 (17)
C4—H4A	0.9500	C17—H17	0.9500
C5—C6	1.398 (2)	C18—C19	1.4742 (15)
C5—H5	0.9500		
			
C1—N1—C8	109.16 (9)	C10—C9—H9A	108.4
C1—N1—C9	124.09 (10)	N1—C9—H9B	108.4
C8—N1—C9	126.44 (10)	C10—C9—H9B	108.4
C19—N3—C20	111.43 (9)	H9A—C9—H9B	107.5
C19—N3—H3	124.3	C11—C10—C15	119.39 (11)
C20—N3—H3	124.3	C11—C10—C9	117.71 (10)
C20—N4—C18	111.14 (9)	C15—C10—C9	122.75 (10)
C20—N4—H4	124.4	C12—C11—C10	120.79 (11)
C18—N4—H4	124.4	C12—C11—H11	119.6
N1—C1—C2	110.27 (10)	C10—C11—H11	119.6
N1—C1—H1	124.9	C11—C12—C13	119.35 (11)
C2—C1—H1	124.9	C11—C12—H12	120.3
C1—C2—C17	128.96 (11)	C13—C12—H12	120.3
C1—C2—C3	105.97 (10)	C14—C13—C12	120.33 (11)
C17—C2—C3	125.00 (10)	C14—C13—C16	119.57 (11)
C4—C3—C8	119.03 (11)	C12—C13—C16	120.10 (11)
C4—C3—C2	133.85 (11)	C15—C14—C13	119.62 (11)
C8—C3—C2	107.12 (10)	C15—C14—H14	120.2
C5—C4—C3	118.39 (12)	C13—C14—H14	120.2
C5—C4—H4A	120.8	C14—C15—C10	120.43 (11)
C3—C4—H4A	120.8	C14—C15—H15	119.8
C4—C5—C6	121.63 (13)	C10—C15—H15	119.8
C4—C5—H5	119.2	N2—C16—C13	178.46 (14)
C6—C5—H5	119.2	C18—C17—C2	129.12 (11)
C7—C6—C5	121.37 (12)	C18—C17—H17	115.4
C7—C6—H6	119.3	C2—C17—H17	115.4
C5—C6—H6	119.3	C17—C18—N4	130.58 (10)
C6—C7—C8	116.92 (11)	C17—C18—C19	124.41 (10)
C6—C7—H7	121.5	N4—C18—C19	104.99 (9)
C8—C7—H7	121.5	O1—C19—N3	126.12 (10)
N1—C8—C7	129.86 (11)	O1—C19—C18	128.34 (10)
N1—C8—C3	107.48 (10)	N3—C19—C18	105.54 (9)
C7—C8—C3	122.66 (11)	O2—C20—N4	127.55 (11)
N1—C9—C10	115.55 (9)	O2—C20—N3	125.55 (11)
N1—C9—H9A	108.4	N4—C20—N3	106.90 (10)
			
C8—N1—C1—C2	−0.07 (13)	C15—C10—C11—C12	3.09 (18)
C9—N1—C1—C2	173.86 (10)	C9—C10—C11—C12	−172.46 (11)
N1—C1—C2—C17	−176.56 (11)	C10—C11—C12—C13	−0.67 (19)
N1—C1—C2—C3	0.49 (13)	C11—C12—C13—C14	−1.89 (19)
C1—C2—C3—C4	−179.90 (13)	C11—C12—C13—C16	177.54 (11)
C17—C2—C3—C4	−2.7 (2)	C12—C13—C14—C15	1.98 (18)
C1—C2—C3—C8	−0.72 (12)	C16—C13—C14—C15	−177.45 (11)
C17—C2—C3—C8	176.48 (10)	C13—C14—C15—C10	0.48 (17)
C8—C3—C4—C5	0.24 (18)	C11—C10—C15—C14	−2.99 (17)
C2—C3—C4—C5	179.34 (12)	C9—C10—C15—C14	172.32 (11)
C3—C4—C5—C6	−0.4 (2)	C1—C2—C17—C18	−4.0 (2)
C4—C5—C6—C7	0.0 (2)	C3—C2—C17—C18	179.49 (11)
C5—C6—C7—C8	0.6 (2)	C2—C17—C18—N4	−0.3 (2)
C1—N1—C8—C7	179.20 (12)	C2—C17—C18—C19	−178.46 (11)
C9—N1—C8—C7	5.45 (19)	C20—N4—C18—C17	−178.60 (12)
C1—N1—C8—C3	−0.40 (12)	C20—N4—C18—C19	−0.22 (12)
C9—N1—C8—C3	−174.15 (10)	C20—N3—C19—O1	179.92 (11)
C6—C7—C8—N1	179.66 (12)	C20—N3—C19—C18	0.11 (12)
C6—C7—C8—C3	−0.79 (18)	C17—C18—C19—O1	−1.22 (19)
C4—C3—C8—N1	−179.99 (10)	N4—C18—C19—O1	−179.74 (11)
C2—C3—C8—N1	0.69 (12)	C17—C18—C19—N3	178.58 (10)
C4—C3—C8—C7	0.38 (17)	N4—C18—C19—N3	0.06 (11)
C2—C3—C8—C7	−178.95 (11)	C18—N4—C20—O2	179.95 (12)
C1—N1—C9—C10	103.89 (13)	C18—N4—C20—N3	0.28 (13)
C8—N1—C9—C10	−83.24 (14)	C19—N3—C20—O2	−179.91 (11)
N1—C9—C10—C11	−157.33 (11)	C19—N3—C20—N4	−0.24 (13)
N1—C9—C10—C15	27.28 (16)		

### Hydrogen-bond geometry (Å, °)

**Table:**

*D*—H···*A*	*D*—H	H···*A*	*D*···*A*	*D*—H···*A*
N3—H3···O1^i^	0.88	1.95	2.8237 (12)	173
N4—H4···O2^ii^	0.88	2.07	2.8740 (13)	151

Symmetry codes: (i) −*x*+3/2, −*y*+1/2, −*z*+1; (ii) −*x*+1, −*y*, −*z*+1.
